# Case for diagnosis. Fibroepithelioma of Pinkus in a 76-year-old patient^⋆^

**DOI:** 10.1016/j.abd.2021.08.009

**Published:** 2022-06-06

**Authors:** Nicole Baldin, Gabriela Galvão Santos, Paulo Ricardo Martins Souza, Laura Luzzatto

**Affiliations:** a Faculty of Medicine, Universidade Franciscana, Santa Maria, RS, Brazil; b Dermatology Outpatient Clinic, Hospital Santa Casa de Misericórdia em Porto Alegre, Porto Alegre, RS, Brazil

## Case Report

**Figure 1:**
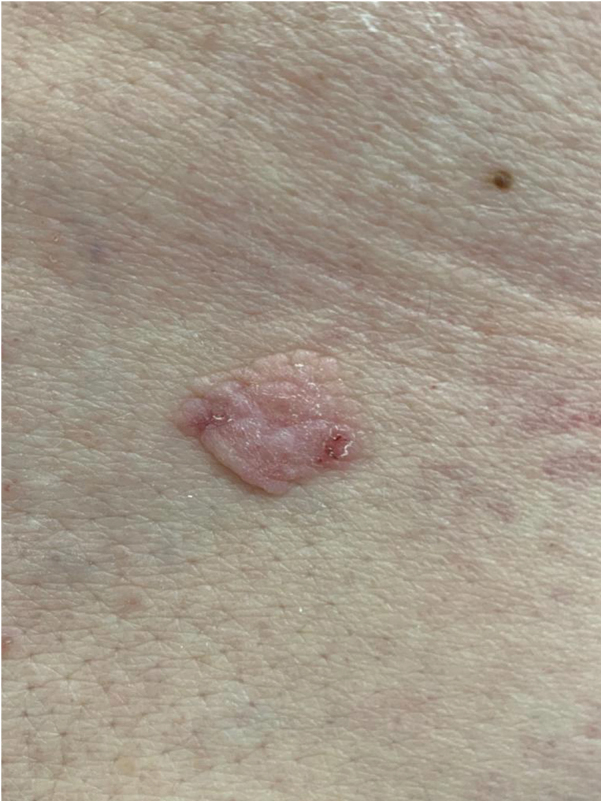
Normochromic papillomatous plaque with a slightly erythematous center.

**Figure 2:**
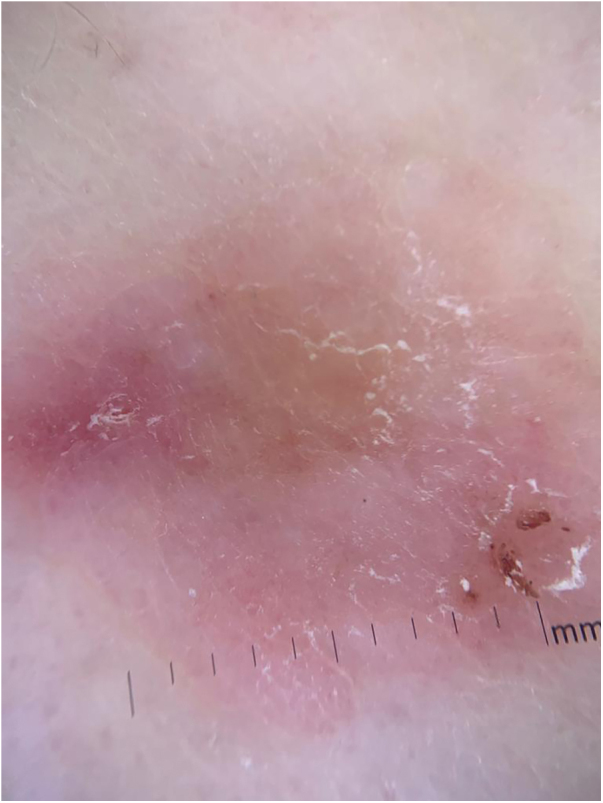
Dermoscopy showing the presence of white striae and microulcerations.

**Figure 3:**
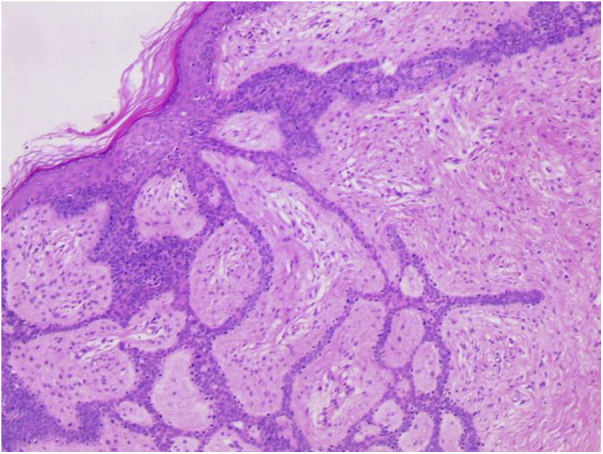
Long, thin, branched and anastomosed epithelial strands of basaloid cells originating from the epidermis embedded in a fibrous stroma. (Hematoxylin & eosin, ×100).

**Figure 4:**
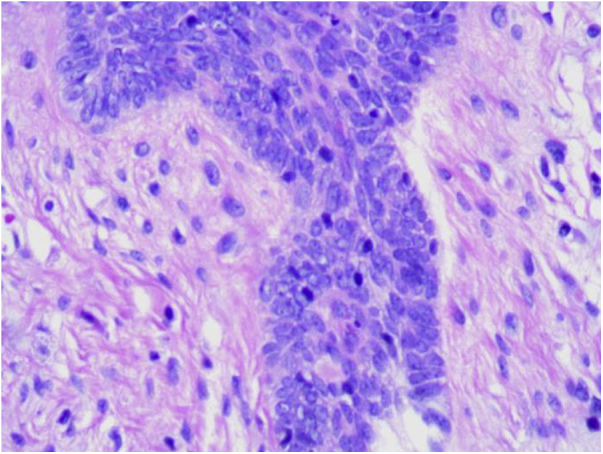
Epithelial cell clusters arranged in palisade can be seen along the epithelium, like “buds on a branch”. (Hematoxylin & eosin, ×400).

A 76-year-old patient complained an asymptomatic lesion for more than 6 months, consisting of a normochromic plaque on the lower back, somewhat papillomatous with a slightly erythematous center (Fig. 1), with peripheral white striae and microulcerations on dermoscopy (Fig. 2). Histopathological examination showed an epithelial basaloid proliferation with a focally reticulate pattern (Figure 3, Figure 4). The excision of the lesion was performed.

## What’s your diagnosis?

a) Seborrheic keratosis
b) Fibroepithelioma of Pinkus
c) Superficial basal cell carcinoma
d) Trichoblastoma

## Discussion

Fibroepithelioma of Pinkus (FeP) is considered an uncommon type of basal cell carcinoma (BCC), first described by Hermann Pinkus in 1953, who named it a premalignant variant of basal cell epithelioma, and similar to trichoblastomas regarding their degrees of differentiation.^1^, ^2^ Therefore, some researchers have claimed that both BCC and trichoblastomas can be better classified as opposing representatives of the same spectrum of differentiation, with FeP deserving an intermediate classification within this fine line.^1^

It is more often reported in females (54%) and the elderly^3^ and is probably underreported. It presents clinically as normochromic/brown single or multiple lesions, such as papules or plaques, dome-shaped or sessile, which can mimic benign skin lesions that would not be routinely excised or biopsied, such as pedunculated fibroma, acrochordon, seborrheic keratosis, and dermal nevus.^4^, ^5^ It affects non-photoexposed areas such as the lumbosacral and abdominal region, groin, and foot. On dermoscopy, thin branched vessels, punctiform vessels, white septal striae, corneal pseudocysts, and ulcerations are observed. Some lesions show structureless gray-brown pigmentation and bluish-gray dots.^6^

Histopathological examination is crucial for the diagnosis as it reveals thin strands of basaloid cells surrounded by stroma, which form a uniform border with the underlying dermis in a fenestrated, honeycomb-like pattern.^7^, ^8^

Treatment consists of the surgical excision of the lesion. The prognosis is good, with low local aggressiveness and low risk for metastasis.^9^

Regarding the above mentioned differential diagnoses, seborrheic keratosis is a benign, rounded, or irregular lesion with a brownish or black color, affecting mainly the face and trunk.^2^ Superficial basal cell carcinoma more commonly affects the face and neck, presents wide branched vessels on dermoscopy, and is less differentiated on histopathology, not fenestrated.^5^ Trichoblastoma is a rarer tumor, with fine arboriform vessels, crown vessels, and pearly white background on dermoscopy, with a fibrocystic stroma, formation of follicular bulbs and papillae on histopathology.^2^, ^8^

## Financial support

None declared.

## Authors’ contributions

Nicole Baldin: Drafting and editing of the manuscript; critical review of the literature; approval of the final version of the manuscript.

Gabriela Galvão Santos: Drafting and editing of the manuscript; critical review of the literature; approval of the final version of the manuscript.

Paulo Ricardo Martins Souza: Drafting and editing of the manuscript; critical review of the literature; approval of the final version of the manuscript.

Laura Luzzatto: Anatomopathological evaluation; intellectual participation in the propaedeutic conduct; approval of the final version of the manuscript.

## Conflicts of interest

None declared.
